# Chimeric antigen receptor (CAR)+ T cell selection method affects transgene efficacy

**DOI:** 10.1186/2051-1426-1-S1-P32

**Published:** 2013-11-07

**Authors:** David Rushworth, Tiejuan Mi, Simon Olivares, Rosa Santana Carrero, Ge Yang, Amer Najjar, Laurence Cooper

**Affiliations:** 1 Pediatrics, MD Anderson Cancer Center, Houston, TX, USA; 2 Graduate School of Biomedical Sciences, UT Houston Health Science Center, Houston, TX, USA; 3 Medical Sciences, University of Puerto Rico School of Medicine, San Juan, US Minor Outlying Islands

## 

The use of CAR+ T cells for the treatment of cancer is growing as multiple centers participate in Phase I/II clinical trials. Prior studies of CAR-dependent T cell effector function evaluated CAR design on T-cell responses in vitro and in vivo. Our study assesses the effect ex vivo co-stimulation imparts on in vitro and in vivo effector function of CAR+ T cells. In this study, the well characterized CD19-specific 2nd generation CAR, signaling through CD28 and CD3-ζ endodomains, was expressed in donor T cells. Non-virally transformed T cells, induced to genomically integrate CAR by Sleeping Beauty transposase, were numerically expanded on artificial antigen presenting cells (aAPC) derived from K562. The aAPC were genetically modified to present the target antigen CD19 along with no co-stimulation, or co-stimulation via CD86, CD137L, or both molecules. The addition of co-stimulation to the culture impacted the expression of CAR and the phenotype of the CAR+ T cells. The co-expression of a second transgene, inducible Caspase 9 (iC9) - a suicide gene, with CAR was also affected by the choice of aAPC. Furthermore, the anti-tumor activity of the CAR+ T cells numerically expanded on aAPC with or without co-stimulation was tested by adoptive transfer into mice containing CD19+ tumor. These data highlight that the use of co-stimulation in the ex vivo culture could potentially impact the therapeutic potential of CAR+ T cells.

